# Electrolyte Gated Transistors for Brain Inspired Neuromorphic Computing and Perception Applications: A Review

**DOI:** 10.3390/nano15050348

**Published:** 2025-02-24

**Authors:** Weisheng Wang, Liqiang Zhu

**Affiliations:** School of Physical Science and Technology, Ningbo University, Ningbo 315211, China; 2011077056@nbu.edu.cn

**Keywords:** artificial intelligence, neuromorphic computing, electrolyte-gated transistors, bionic synapses, artificial perceptual systems

## Abstract

Emerging neuromorphic computing offers a promising and energy-efficient approach to developing advanced intelligent systems by mimicking the information processing modes of the human brain. Moreover, inspired by the high parallelism, fault tolerance, adaptability, and low power consumption of brain perceptual systems, replicating these efficient and intelligent systems at a hardware level will endow artificial intelligence (AI) and neuromorphic engineering with unparalleled appeal. Therefore, construction of neuromorphic devices that can simulate neural and synaptic behaviors are crucial for achieving intelligent perception and neuromorphic computing. As novel memristive devices, electrolyte-gated transistors (EGTs) stand out among numerous neuromorphic devices due to their unique interfacial ion coupling effects. Thus, the present review discusses the applications of the EGTs in neuromorphic electronics. First, operational modes of EGTs are discussed briefly. Second, the advancements of EGTs in mimicking biological synapses/neurons and neuromorphic computing functions are introduced. Next, applications of artificial perceptual systems utilizing EGTs are discussed. Finally, a brief outlook on future developments and challenges is presented.

## 1. Introduction

The rapid development of information technologies, such as artificial intelligence (AI), cloud computing, and Internet of Things (IoT), has propelled human society toward greater intelligence [1]. However, this has also resulted in an explosive increase in global data volume, creating higher demands for the fast and efficient processing of information. On the other hand, the transmission and storage of large volumes of data still rely on traditional von Neumann architecture. The end of Moore’s Law and the von Neumann bottleneck would result in challenges, including limited parallel processing, high energy consumption, and increased latency in computing [2,3]. To meet the growing demands for data storage and information processing, there is an urgent need to develop new devices and innovative computing paradigms. Brain inspired neuromorphic computing is a computational paradigm designed to simulate the structure and function of a human brain [4]. It achieves functionalities similar to biological neurons and synapses through hardware implementation, offering advantages such as parallel processing, event-driven operation, and low energy consumption, thereby providing a new paradigm for addressing such challenges.

Neuromorphic devices can mimic neural and synaptic functions, posing great potentials in hardware-based neuromorphic platforms [5,6]. Among various neuromorphic devices, ionic–liquid, ionic–gel and solid-state ionic–liquid based electrolyte-gated transistors (EGTs) have been deemed as ideal components for bionic neuromorphic electronic applications [7,8,9]. Such devices have unique capacitive coupling mode, exhibiting flexible memory effects and diverse synaptic plasticity. Furthermore, their characteristics, such as the separated reading/writing operation modes, tunable ionic dynamics timescales, and multi-gate configurations, render them a robust design solution for high-efficiency, low-power neuromorphic circuits. Leveraging the unique ionic coupling characteristics of EGTs, a series of neuromorphic functions have been simulated successfully, including short-term and long-term synaptic plasticity [10,11,12], synaptic learning rules [13,14], conditioned reflexes [15,16], and pattern recognition [17,18]. Meanwhile, the human perceptual system is a complex multimodal collaborative learning system [19,20]. The integration of visual, tactile, auditory, olfactory and gustatory inputs within the multisensory neural network enhances higher cognitive functions such as information integration, recognition, reasoning, and imagination [21]. Therefore, hardware level simulation of bio-inspired sensory functions is also of extraordinary significance [22]. Thus, integration of EGTs with diverse artificial receptors has been reported, enabling successful demonstration of EGT-based bio-inspired perceptual systems including vision [23,24], tactile sensing [25,26], audio-visual fusion [27], etc. These advances outline novel design strategies for constructing artificial perceptual platforms. However, it should be noted here that brain-inspired computing and bionic perception based on EGTs remain in their infancy. A comprehensive understanding of the current research progress and challenges is essential to advance the field.

In this review, we briefly summarize the research progress on neuromorphic function simulation and artificial perceptual system applications based on EGTs. First, we introduce EGTs and discuss their operational mechanisms. Next, we summarize the use of EGTs for implementing synaptic and neural functions as well as neuromorphic computing. Finally, we discuss multifunctional intelligent artificial perceptual systems constructed by integrating EGTs with different bionic sensors. At the end, a brief outlook on future developments and challenges is made.

## 2. Electrolyte-Gated Transistors (EGTs)

**Figure 1:**
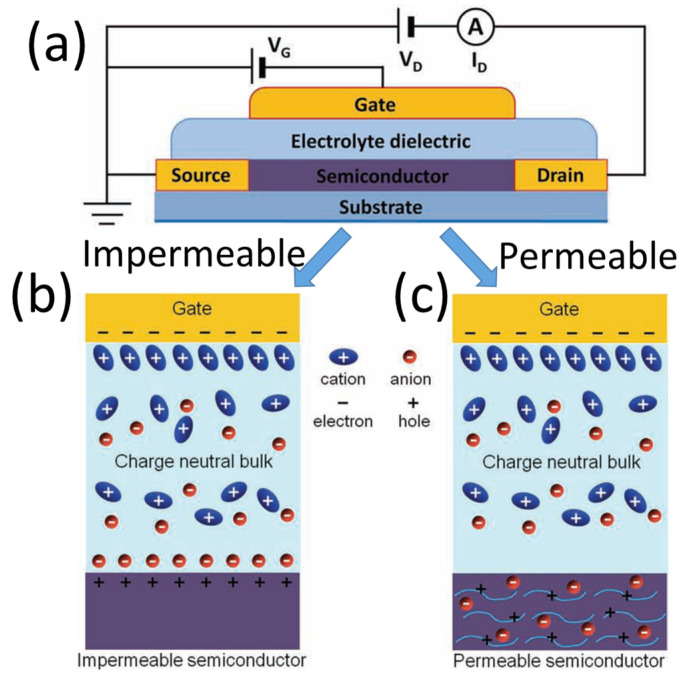
(**a**) Cross-section image of an EGT. (**b**) Schematic representation of the electrostatic modulation operating mode, i.e., EDLTs. (**c**) Schematic representation of the electrochemical doping operating mode, i.e., ECTs. Reproduced with permission [31]. Copyright 2013, WILEY-VCH Verlag GmbH & Co. KGaA, Weinheim, Germany.

The history of EGTs can be traced back to the 1950s [28]. EGTs have a structure similar to conventional field effect transistors. The difference is that EGTs adopt electrolytes as gate dielectrics. EGTs exhibit superior ionic/electronic coupling behavior and distinctive ion relaxation dynamics, enabling the formation of extremely high electric fields, large capacitances and elevated carrier concentrations. This results in significantly reduced power consumption. Figure 1 schematically shows the EGTs. Under an external electric field, ions within an electrolyte migrate toward the electrolyte/channel interface and accumulate there. Due to electrostatic coupling, an equivalent number of charge carriers with opposite polarity will accumulate on the channel side, ultimately forming an electric-double-layer (EDL). Ions might penetrate into the channel through electrochemical reactions, resulting in electrochemical doping in the channel. Benefiting from the high coupling efficiency between gate and channel, EGTs operate at very low voltages and can accommodate multi-gate inputs, making them a powerful design solution for efficient, low-power neuromorphic circuits. Furthermore, EGTs exhibit tunable ionic dynamic time scales, suitable for simulating dynamical characteristics of biological neural systems [29,30]. Notably, most electrolyte materials serving as gate dielectrics can be processed at room temperature via solution-based methods, facilitating their integration into neuromorphic electronics and flexible electronics applications. Thus, EGTs can serve as switching components, artificial synapses, artificial neurons, and memristive systems, enabling the development of compact and robust neuromorphic computing networks.

### 2.1. Operation Modes of EGTs

EGTs operate by modulating the migration of ions within an electrolyte, which enables the regulation of carrier concentrations in channels, ultimately achieving transistor switching functionality. Based on whether ions within electrolyte can penetrate into the semiconductor channel layer under an external electric field, EGTs operate in two distinct modes, i.e., electrostatic modulation and electrochemical doping [31,32], as schematically illustrated in Figure 1.

#### 2.1.1. Electrostatic Modulation

In electrostatic modulation mode, dissociated cations and anions within the electrolyte will migrate and accumulate at the electrolyte/channel or electrolyte/gate interface under an external electric field. This process induces a layer of high-concentration charge carriers with opposite polarity and equal total charge on either the channel or the gate electrode. Thus, an EDL layer will form at the electrode/electrolyte or channel/electrolyte interface, as schematically shown in Figure 1b. Under steady-state conditions, the electric field is predominantly confined within the EDL layer, with minimal potential drop across the whole electrolyte. The EDL, characterized with an ultrathin thickness of ~10^−9^ m, exhibits an exceptionally high specific capacitance (>1 μF/cm^2^), far exceeding that of conventional gate dielectrics in thin-film transistors. This distinctive EDL effect at the electrolyte/channel interface underpins the designation of electric-double-layer transistors (EDL-Ts). Benefiting from strong electrostatic modulation and ionic relaxation, EDL-Ts operate at low voltages (<2 V) and exhibit volatile conductance changes in the channel. These attributes align well with short-term synaptic plasticity in biological systems. Thus, EDL-Ts exhibit exceptional features, including low operating voltage, high current modulation capability, fast response speed, and low power consumption, making them highly attractive for neuromorphic applications.

#### 2.1.2. Electrochemical Doping

In electrochemical doping mode, anions/cations within an electrolyte will accumulate at the electrolyte/channel interface under an external electric field. They will also penetrate into a semiconductor channel, inducing electrochemical doping [33], as schematically shown in Figure 1c. Devices exploiting this mechanism are termed as electrochemical transistors (ECTs). ECTs can undergo not only electrochemical doping but also de-doping processes under reverse bias. Fundamentally reliant on these reversible doping/de-doping mechanisms, such devices induce nonvolatile modifications of channel conductivities. These inherent characteristics position them as promising candidates for emulating long-term plasticity of synapses in synaptic electronics. ECTs achieve channel conductivity modulation by driving ion insertion into the channel layer to alter its redox state, endowing them with remarkable attributes including high transconductance, low operating voltage, fast switching speeds, and high sensitivity. These combined merits endow them with significant potential as next-generation neuromorphic devices.

### 2.2. Electrolytes for EGTs

Electrolyte-gated transistors, with rich ionic dynamics, low power consumption, and information processing advantages, have been widely applied to simulate synaptic plasticity behaviors and advanced neuromorphic functions of the human brain. From the perspective of ion migration, different electrolytes have different hindrance and promotion effects on ion migration, which will lead to different stability and electrical properties of EGTs. Therefore, the development of composite electrolytes will significantly broaden the applications of EGTs in various fields. By precisely aligning material properties with neuromorphic functional requirements, EGTs hold significant potential for breakthroughs in brain-inspired computing, sensing-computing integration, and adaptive systems. Researchers have developed electrolyte materials tailored for diverse application scenarios to achieve effective modulation of EGTs, including polymer electrolytes, ionic liquids, ion gels, and inorganic solid-state electrolytes [34,35,36,37,38]. These materials enable precise control over device performance through optimized ionic/electronic interactions, environmental adaptability, and interface engineering.

#### 2.2.1. Polymer–Electrolytes

**Figure 2:**
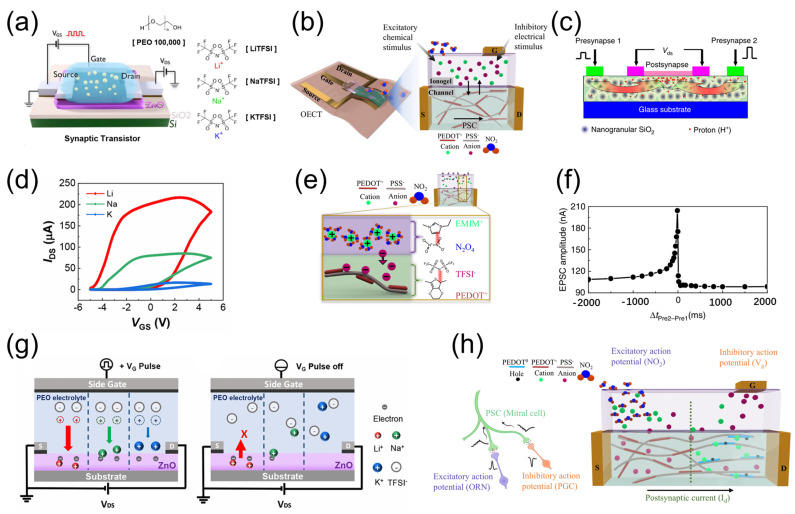
(**a**) Schematic illustration of alkali ion containing poly(ethylene oxide) (PEO) electrolyte-gated transistors. (**b**) The memory window of AGT under different ion species. (**c**) The effect of gate pulse on the ion-specific long-term memory mechanism at the ZnO/PEO electrolyte interface. Reproduced with permission [42]. Copyright 2024, American Chemical Society. (**d**) Schematic illustration of the organic electrochemical transistor gated by the potential generated by the interaction of gas molecules with ions in a chemoreceptive ionogel. (**e**) Artificial chemical synapses with the capability to respond to chemical stimuli. (**f**) Simulation of excitatory and inhibitory synaptic functions in the olfactory system. Reproduced with permission [47]. Copyright 2023, Springer Nature. (**g**) Schematic illustration of the nanogranular SiO_2_-gated IZO synaptic transistor with two in-plane gates. (**h**) Spatiotemporal dynamic logic simulation of laterally coupled synaptic transistors based on two in-plane gates. Reproduced with permission [52]. Copyright 2014, Springer Nature Limited, London, UK.

Polymer–electrolytes are formed by dissolving a salt in polymer. The conducting mechanism involves the segmental motion-assisted diffusion of the ion within the polymer matrix. Generally, they are bendable, compatible with flexible substrates, and easy to process. Poly (ethylene oxide) (PEO)/perchlorate (AClO_4_, A = Li, K, etc.) is a common type of polymer–electrolyte. The oxygen lone pair electrons on the PEO chain are paired with metal cations. Due to the flexibility of the PEO chain, metal cations can couple ion migration on the PEO backbone chain. Frisbie et al. [39] observed a very large carrier density in the order of 10^15^/cm^2^ in poly-3-hexylthiophene (P3HT) using a PEO/LiClO_4_ based electrolyte. Zou et al. [40] proposed a PEO/LiClO_4_ polymer electrolyte gated field effect transistor with a single SnO_2_ nanowire channel to simulate synaptic function. Thanks to the excellent ion/electron interface coupling effect of PEO/LiClO_4_ polymer electrolytes, the device achieves excellent carrier mobility with a maximum mobility of ~64.1 cm^2^/Vs. Wang et al. [41] reported a laterally coupled 2D MoS_2_ synaptic transistor device utilizing a PEO/LiClO_4_ ion-conducting electrolyte. Due to the strong EDL effect, a low operating voltage of 1 V and a high current on/off ratio of 10^5^ have been obtained. As shown in Figure 2a, Lee et al. [42] conducted an electrochemical analysis of PEO EGTs (AGTs) containing alkali metal cations (Li^+^, Na^+^, and K^+^) combined with bis(trifluoromethanesulfonyl)imide (TFSI^−^) anions to systematically investigate the effects of ion species on synaptic performance. Figure 2b displays double-scan transfer curves of AGTs, showing counterclockwise hysteresis. However, compared to other AGTs, Li-AGTs exhibit a larger memory window. This is attributed to the smaller ionic radius of Li^+^, allowing it to occupy deeper interstitial or substitutional sites in ZnO. Additionally, to better understand the impact of ionic size on electrochemical performance, that work conducted electrochemical analyses of interfacial reactions. As shown in Figure 2c, the smaller Li^+^ can be easily doped into the ZnO electrode. Its higher mobility in the electrolyte facilitates Faradaic reactions upon the application of bias. The systematic electrochemical analysis with a clear mechanism provides new insights for the selection of high-performance artificial synaptic materials. Polymer electrolytes exhibit exceptional flexibility and tunable ionic conductivity, making them highly attractive for flexible electronics. Their design versatility enables tailored combinations of polymer matrices and conductive lithium salts with adjustable ratios to meet diverse application requirements. However, the limited ion migration rate along polymer chains results in a slow response to gate voltages, restricting their application in high-frequency devices. Additionally, challenges such as insufficient cycling durability and difficulties in controlling film uniformity hinder their performances in long-term stability and miniaturization. Future advancements may focus on composite electrolyte design and interface engineering, which could unlock broader applications in emerging technologies.

#### 2.2.2. Ionic Liquids or Ion Gel

Ionic liquids (ILs), a class of liquids entirely composed of cations and anions, enable diverse compound formations through various combinatorial approaches. Their superior ionic conductivity, enhanced switching speed, and faster operating frequency establish ILs as preferable electrolyte materials for EGTs [31,43]. Yuan et al. [44] reported an EDL-T utilizing ionic liquid (DEME-TFSI) as a gate dielectric, achieving ultra-high-density electron accumulation, i.e., up to 4.5 × 10^14^ cm^−2^ at room temperature. Notably, this ionic liquid-based gate dielectric exhibited charge accumulation capability even at low temperatures, reaching an extraordinarily high carrier density of 8 × 10^14^ cm^−2^ at 220 K and maintaining a density of 5.5 × 10^14^ cm^−2^ at 1.8 K. That work provides a new strategy for realizing devices with high carrier densities. However, the inherent liquid nature of ionic liquids may limit their practical applications in complex scenarios.

Consequently, ion gels, which serve as solid electrolytes, have been proposed as alternatives. Ionic gels are typically formed by dissolving block copolymers in ionic liquids and undergoing gelation. They not only retain the advantages of ionic liquids but also possess desirable characteristics like good physical/chemical stability, flexibility, lightweight, and transparency [45,46]. Chouhdry et al. [47] presented an organic electrochemical transistor with a poly(3,4-ethylenedioxythiophene) polystyrene sulfonate (PEDOT:PSS) channel and an ionogel (IG) based on 1-ethyl-3-methylimidazolium bis(trifluoromethylsulfonyl)imide ([EMIM] [TFSI]) ionic liquid (IL), poly(ethylene glycol) diacrylate (PEGDA) monomer, and a1-hydroxycyclohexyl phenyl ketone photoinitiator as a chemoreceptive material as well as gate electrolyte layer, as shown in Figure 2d. Thanks to the excellent ionic dynamics of the ionogel gate electrolyte, the device demonstrates long-term memorization to excitatory chemical stimulus. Interestingly, this system can also simulate the chemical synaptic functions in biological olfactory systems, as illustrated in Figure 2e. Upon exposure to NO_2_, the gas molecules interact with cations in IG, driving ions into the channel in a neurotransmitter-like manner, thereby modulating the postsynaptic current (PSC). Furthermore, by applying chemical stimulation and electrical inhibition, the ion gel gated ECT can simulate both excitatory and inhibitory synaptic functions in the olfactory system (Figure 2f). This advancement further contributes to the development of artificial neuron systems for bionic chemosensory applications. Notably, ILs demonstrate ultrahigh ionic conductivity and wide electrochemical stability windows, enabling low-voltage operation and precise signal modulation. However, their liquid nature raises leakage risks and exhibits poor mechanical stability, limiting their flexible applications. In contrast, ion gels exhibit superior physical/chemical stability coupled with flexibility and optical transparency. Current challenges in large-scale ion gel processing persist. Conventional methods, such as spin-coating and printing, risk contaminating semiconductor layers. Future research would focus on optimizing ion gel formulations to improve interfacial compatibility, mechanical robustness, and electrical performance, thereby enabling their practical implementation across diverse fields.

#### 2.2.3. Inorganic Solid-State Electrolytes

Inorganic solid-state electrolyte-gated EGTs are compatible with standard fabrication processes and can be produced at low temperature, attracting significant interest in the field of neuromorphic electronics. In comparison to polymer electrolytes and IL electrolytes, solid-state electrolytes exhibit excellent chemical stability and high ionic conductivity [23,48,49,50]. Nanogranular SiO_2_ film can be deposited at room temperature using silane and oxygen as reactant gases through plasma-enhanced chemical vapor deposition (PECVD). It can act as a novel inorganic solid-state electrolyte film. In 2009, Jiang et al. [51] prepared a nanogranular SiO_2_ solid electrolyte film with an EDL capacitance exceeding 1 μF/cm^2^. It served as the basis for developing ultralow-voltage transparent InGaZnO_4_ EDL-T. In 2014, Zhu et al. [52] proposed IZO synaptic transistor based on phosphorus (P)-doped nanogranular SiO_2_ proton conductive films, as illustrated in Figure 2g. Thanks to the strong proton-related EDL effect of the SiO_2_ thin film, effective modulation of the channel conductivity can be achieved through a lateral gate coupling effect. Furthermore, spatiotemporal dynamic logic functions were implemented under synergistic regulation of two in-plane gates, as shown in Figure 2h. Additionally, they developed a simple solution-processed sodium alginate proton conductor film with a large EDL capacitance of up to 2.0 µF/cm^2^ [53]. Using sodium alginate proton conductor film as a gate dielectric, the transistor exhibited a current switching ratio of 3.1 × 10^6^, an operating voltage of below 1.5 V, a subthreshold swing of 100 mV/decade, and a high electron mobility of 8.8 cm^2^/V·s. These findings demonstrate the potential of using solid-state electrolytes as gate dielectrics in low-power portable electronics, including high ionic conductivity, robust mechanical strength, and excellent chemical stability. Doping serves as the predominant strategy to enhance their ionic conductivity. These electrolytes can operate effectively at temperatures compatible with conventional electronics, positioning them as promising candidates for neuromorphic electronics. However, the interfacial challenges of high contact resistance between inorganic solid-state electrolytes and electrodes may adversely affect transistor performances.

### 2.3. Channels for EGTs

**Figure 3:**
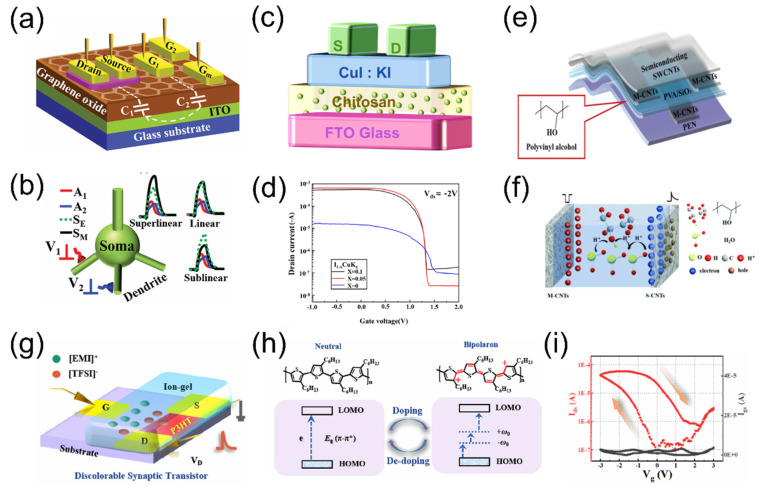
(**a**) Schematic diagram of multi-gate IZO-based neuron transistor. (**b**) Schematic of nonlinear dendritic integration. Reproduced with permission [66]. Copyright 2016, WILEY-VCH Verlag GmbH & Co. KGaA, Weinheim, Germany. (**c**) Schematic of Cu_0.95_K_0.05_I_x_ EGT. (**d**) Transfer curves of Cu_0.95_K_0.05_I_x_ EGT at different doping concentrations. Reproduced with permission [67]. Copyright 2024, AIP. (**e**) Schematic diagram of flexible transparent carbon nanotube synaptic transistors (F-CNT-STs). (**f**) Mechanism analysis of F-CNT-STs. Reproduced with permission [68]. Copyright 2021, The Royal Society of Chemistry. (**g**) Schematic of the EGT device based on P3HT film. (**h**) Mechanism of ion doping and dedoping of P3HT film under voltage modulation. (**i**) Transfer characteristic curve of EGT devices. Reproduced with permission [75]. Copyright 2023, American Chemical Society, Washington, DC, USA.

Electrical performances of EGTs also depend on the channel materials. Common channel layer materials used in EGTs include metal oxides, low-dimensional materials, and organic materials, etc. [54,55,56,57]. Metal oxide semiconductors stand out as potential candidate channel materials for artificial synaptic transistors due to their high charge carrier mobility, excellent optical transparency, good stability, and suitability for large-area fabrication. The metal oxides used for EGTs include IZO [58,59], IGZO [60,61,62], Ga_2_O_3_ [63], ITO [64], and IWO [65], etc. As shown in Figure 3a, Wan et al. [66] proposed a proton-conducting graphene oxide (GO) film as an electrolyte to prepare a multi-gate IZO-based neuron transistor. Leveraging the strong proton gating effect, this work explored the parallel computing capabilities of simulated neural networks, successfully achieving dendritic integration functions and their modulation, as illustrated in Figure 3b. Although n-type metal oxide channels in synaptic transistors have been extensively studied, p-type metal oxide semiconductors still exhibit deficiencies in electrical performance, primarily due to inherent defects such as localized hole transport pathways in the valence band maximum (VBM) and strong self-compensation effects during doping processes. Consequently, the search for p-type semiconductor materials with excellent hole transport properties and low-temperature synthesis techniques has become a research hotspot. As shown in Figure 3c, Lei et al. [67] fabricated a low-voltage EDL p-type thin-film transistor (TFT) on a glass substrate using copper iodide doped with potassium iodide (Cu_0.95_K_0.05_I_x_) as the channel and chitosan as the dielectric. This was also adopted for neuromorphic system applications. Figure 3d displays the transfer curve at different doping concentration. The KI enhances the transport properties of CuI transistors by increasing the carrier concentration and mobility. This work demonstrates that CuI exhibits high intrinsic Hall mobility, significant p-type conductivity, and strong doping capability, making it one of the most promising candidate materials for channel layers in EGTs.

Additionally, low-dimensional materials, represented by one-dimensional materials such as carbon nanotubes (CNTs) [68] and nanowires [40,69,70,71], as well as two-dimensional materials like black phosphorus [72], molybdenum disulfide (MoS_2_) [38,73], and graphene [74], exhibit exceptional electrical, optical, magnetic, thermal, and mechanical properties due to surface effects, size effects, and quantum tunneling effects. These materials hold significant potential in the fabrication of EGTs. As shown in Figure 3e, Wang et al. [68] introduced a transparent flexible CNT synaptic transistor gated with PVA/SiO_2_ hybrid proton-conducting electrolyte. By effectively modulating mobile ions in the PVA/SiO_2_ mixed dielectric layer, the conductance of the CNT channel can be correspondingly regulated, as shown in Figure 3f. This device features a low operating voltage, fast response speed, and ultra-low power consumption, showing broad prospects in flexible neuromorphic applications. Compared to other materials, organic materials possess designable processing characteristics, simple fabrication techniques and good compatibility with flexible substrates. These features have led to their widespread use in EGTs. Common representative organic materials include pentacene, poly(3-hexylthiophene) (P3HT), poly(3,4-ethylenedioxythiophene):polystyrene sulfonate (PEDOT:PSS), and poly(3,4-ethylenedioxythiophene) doped with poly(tetrahydrofuran) (PEDOT:PTHF). Among these, P3HT is a typical p-type semiconductor used as a channel material and is widely regarded as an attractive high-mobility hole transport material for various organic electronic devices. As shown in Figure 3g, Sun et al. [75] fabricated a flexible multifunctional EGT using [EMI][TFSI]-based ion-gel film as a gate dielectric and P3HT film as the channel material. Interestingly, through appropriate voltage programming, the P3HT-based EGTs exhibit simultaneous modulation of electronic conductivity and light absorption properties, making them ideal materials for achieving multifunctional discolorable EGT devices, as shown in Figure 3h. Furthermore, the device exhibits excellent memory and conductivity modulation characteristics. Figure 3i presents the transfer characteristic curve of the device, showing a notable hysteresis window with an approximately 100-fold increase in conductivity. This device has significant implications for the design of electrolyte-gated synaptic devices and multifunctional systems. However, devices based on organic semiconductors still face significant challenges in large-scale production and performance stability. The structural inhomogeneity of organic polymers can lead to considerable fluctuations in device performance when the device size is reduced to the nanoscale, ultimately affecting its stability and reliability.

## 3. Implementation of Neuromorphic Functions on EGTs

### 3.1. Synaptic Functions

**Figure 4:**
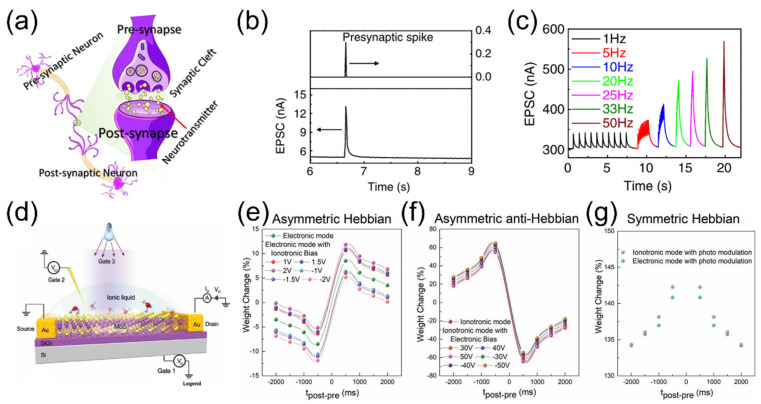
(**a**) Schematic diagram of a biological synaptic structure. Reproduced with permission [85]. Copyright 2023, Elsevier Ltd., Kidlington, UK (**b**) Typical EPSC response triggered by 0.3 V, 10 ms. (**c**) EPSCs recorded in response to the stimulus train with different frequencies. Reproduced with permission [52]. Copyright 2014, Springer Nature Limited., London, UK (**d**) Schematic diagram of the MoS_2_ EGTs in multi-signal operating modes (electrical, ionic, and optical signals). (**e**) Modulation of STDP behavior in electrical signal mode by ionic signals. (**f**) Modulation of STDP behavior in ionic signal mode by electrical signals. (**g**) Symmetric STDP behavior observed under optical mode. Reproduced with permission [84]. Copyright 2018, WILEY-VCH Verlag GmbH & Co. KGaA, Weinheim, Germany.

Synapses are the critical nodes between neurons for functional connectivity and information transmission. They not only allow neural signals to pass from one neuron to another but also play a key role in information processing, learning, and memory formation. As shown in Figure 4a, a biological synapse consists of a presynaptic membrane, synaptic cleft, and postsynaptic membrane. When an action potential reaches the presynaptic membrane, it triggers the exocytosis of synaptic vesicles. The released neurotransmitters interact with various receptors on the postsynaptic membrane, activating ion channels [76,77]. This leads to the generation of a new action potential, thereby transferring the signal from the presynaptic neuron to the postsynaptic neuron. Bionic neurons and bionic synapses can transmit electrical signals and convert them into neural-like signals, which is expected to achieve compatibility with biological neural signals and be used to build intelligent and efficient artificial intelligence. The EGTs combine electrostatic gating and electrochemical doping in a single device, where the ion motion triggered by electrical inputs can induce both volatile and non-volatile modulation of the channel conductance. These properties have been demonstrated to be highly suitable for emulating synaptic responses and learning behaviors of biological neurons.

In recent years, numerous EGTs have been reported for applications in synaptic bionics and neuromorphic engineering, mimicking rich neuromorphic functions [10,11,12,13,14,78,79,80], such as short-term/long-term plasticity, synaptic filtering, spiking-timing dependent plasticity (STDP), spiking rate dependent plasticity (SRDP), and Bienenstock–Cooper–Munro (BCM) learning rules, etc. Zhu et al. [52] proposed an IZO synaptic transistor based on phosphorus (P)-doped nanogranular SiO_2_ proton conductive films. The gate voltage can be directly coupled to the IZO channel layer through a lateral proton-related EDL capacitor without the help of a bottom conductive layer, enabling effective modulation of the channel conductivity. The coplanar gate and self-assembled IZO channel, source and drain electrodes can be regarded as the presynaptic and postsynaptic terminals, respectively. Figure 4b shows the typical excitatory postsynaptic current (EPSC) current curve of the synaptic device. By applying a positive voltage spike (0.3 V, 10 ms) to the coplanar gate, protons in the electrolyte can migrate and accumulate at the SiO_2_/IZO interface within a few milliseconds while simultaneously inducing electrons to accumulate at the interface in the channel. Thus, an EPSC response with a peak value of ~13 nA can be detected. When the voltage spike ends, the protons migrated at the interface will diffuse back to their original equilibrium positions, and the EPSC response will gradually decrease to its initial level. This device was also used to mimic a series of short-term plasticity behaviors, including paired pulse facilitation and high-pass filtering. Figure 4c shows the EPSC responses with the decreased spike interval times, i.e., the increased frequencies. Notably, the laterally coupled synaptic transistor is readily scalable to multiple input gates, making construction of synaptic interaction functions convenient. This laterally coupled IZO transistor based on proton-conducting electrolytes shows significant potential in the fields of synaptic electronics and neuromorphic engineering. Long-term synaptic plasticity is widely regarded as one of the major molecular mechanisms underlying learning and memory. Burgt et al. [81] reported an electrochemical neuromorphic organic transistor made from inexpensive, commercially available plastic materials. The device delivers significant non-volatile conductance modulation at low operating voltages of only ~1 V, stable multi-conductance states (>500) and extremely low energy dissipation (<10 pJ for 10^3^ μm^2^ devices). Moreover, the transferring of such artificial synapse devices to PET substrates strongly demonstrates their great potential in flexible electronic systems.

Metaplasticity, first proposed by Abraham, refers to “the plasticity of synaptic plasticity” [82]. It is a higher-order form of synapticity that emphasizes the regulation of synaptic plasticity by introducing a priming stimulus before the main synaptic stimulus. It affects the ability of synapses to produce plasticity [83]. Metaplasticity can be mimicked on EGTs. As shown in Figure 4d, John et al. [84] demonstrated the modulation of STDP behavior along with LTP and LTD behaviors in two-dimensional MoS_2_ EGTs devices using an ionic liquid as a gate dielectric. In contrast to the metaplasticity dependent on pre-synaptic stimulation in memristor devices, this neuromorphic transistor utilizes a multi-gate input structure to achieve multiple operating modes, including electrical, ionic and optical signals. It regulates internal mechanisms through these multi-signal operating modes, maintaining synaptic weights within a dynamic range, thereby enabling metaplastic control over synaptic plasticity. For instance, the STDP behavior in electrical signal mode can be effectively modulated by ionic gate bias, and vice versa, as illustrated in Figure 4e,f. Moreover, the STDP behavior in both electrical and ionic signal modes can also be modulated by light, resulting in symmetric Hebbian STDP, as shown in Figure 4g. The coexistence of various forms of synaptic plasticity enhances the capacity of artificial neurons for memory storage and processing, thereby enabling the design of efficient new neural architectures.

### 3.2. Neural Functions

**Figure 5:**
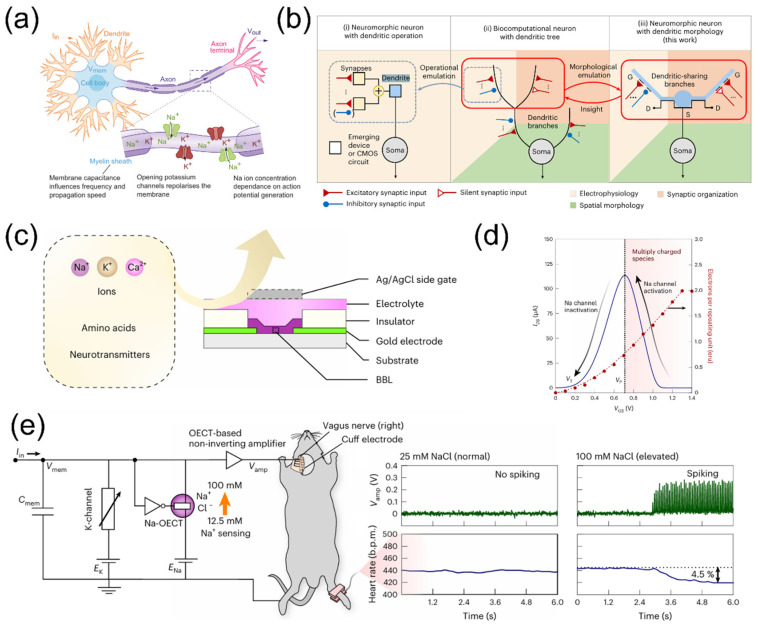
(**a**) Schematic diagram of a biological neuron. Reproduced with permission [86]. Copyright 2022, Springer Nature, London, UK. (**b**) Neuromorphic dendrite model representation. Reproduced with permission [95]. Copyright 2024, Springer Nature. (**c**) Schematic diagram of an organic electrochemical neuron device. (**d**) The antiambipolar behavior of BBL channel material is similar to the activation and inactivation states of voltage-gated sodium channels in the Hodgkin–Hux neuron model. (**e**) c-OECN-based heart rate monitoring in a mouse. Reproduced with permission [96]. Copyright 2023, Springer Nature, London, UK.

Neurons, also known as nerve cells, have a structure as schematically shown in Figure 5a [86]. Neurons can be divided into a cell body (soma) and neurites. Neurites can further be divided into axons and dendrites. Dendrites are short and highly branched, forming synapses with the axons of preceding neurons to receive neurotransmitters. The soma integrates all synaptic information, while the axon generates action potentials and transmits signals to the post neuron [87]. Research indicates that there are over 20 types of firing patterns in neurons, exhibiting rich and complex dynamic characteristics [88]. To fully understand the dynamic behavior of biological neurons, various mathematical models have been proposed, primarily including the Hodgkin–Huxley (HH) neuron model [89,90], the leaky integrate-and-fire (LIF) neuron model [91,92], and oscillatory neuron models [93,94], etc. The human brain contains ~10^11^ neurons, and each can connect with thousands of other neurons through synapses. These neurons and synaptic are organized in three-dimensional space, forming a complex spatiotemporal information processing network, which facilitates the efficient functioning of the human brain. Notably, individual neuron dendrites have been shown to possess spatiotemporal information decoding capabilities, significantly enhancing the information processing capacity of dendrites and improving the efficiency of such networks. Therefore, the development of new concept neuromorphic devices with spatiotemporal information processing functions is crucial for constructing low-power brain-like artificial neural networks (Figure 5b) [95].

Inspired by biological nerve systems, neuromorphic sensing and processing represent a promising strategy for developing bionic devices capable of accurately detecting and rapidly responding to dynamic external stimuli. As shown in Figure 5c, Harikesh et al. [96] introduced a biorealistic conductance-based organic electrochemical neuron (c-OECN) using a mixed ion-electron conducting ladder-type polymer with stable ion-tunable antiambipolarity. As a channel material for organic electrochemical transistors (OECTs), the rigid conjugated polymer, ladder-type poly(benzimidazobenzophenanthroline) (BBL), exhibits a unique, stable, and reversible antiambipolar behavior, which is very similar to the activation and inactivation states of voltage-gated sodium in the HH neuron model, as shown in Figure 5d. Interestingly, these c-OECNs can spike at bioplausible frequencies nearing 100 Hz, replicate most neural features and exhibit stochastic responses in the presence of noise. The BBL-based OECTs possess highly tunable, stable, and reversible antiambipolar characteristics, which hold great potential in applications such as sensing, integration, and neural stimulation. The team successfully monitored the heart rate of a mouse, as shown in Figure 5e, by connecting the c-OECN to the right cervical vagus nerve of the mouse using a cuff electrode. Furthermore, this work demonstrates the ability to achieve event-based sensing by simply adjusting the voltage in the circuit, allowing the c-OECN to respond to specific concentrations of biochemical signals. These results provide innovative insights for the development of future feedback neuromorphic biomedical systems and brain–machine interfaces.

**Figure 6:**
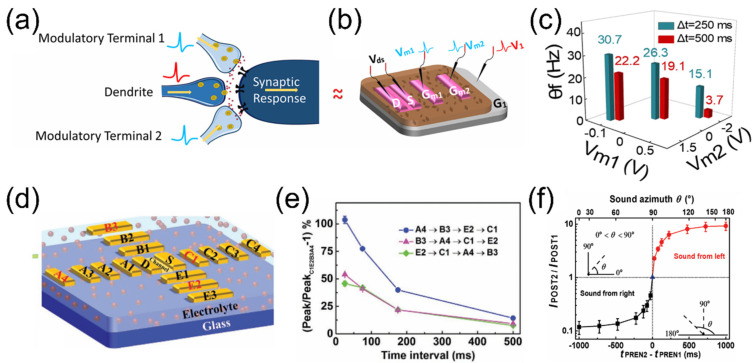
(**a**) Schematic of a heterogeneous synapse with multiple input terminals. (**b**) Schematic of an ITO hetero-dendritic neuron. (**c**) Dynamic regulation of the BCM learning rule by excitatory and inhibitory modulatory stimuli. Reproduced with permission [97]. Copyright 2024, The Royal Society of Chemistry, Cambridge, UK. (**d**) Schematic illustration of an oxide-based neuro-transistor with multiple in-plane gate electrodes in various directions. (**e**) Simulation of the spatiotemporal input sequence recognition function in multiple dendritic branches. (**f**) Detection of sound azimuth in capacitively coupled multiterminal oxide-based neuro-transistor. Reproduced with permission [98]. Copyright 2019, WILEY-VCH Verlag GmbH & Co. KGaA, Weinheim.

Figure 6a shows a schematic of a heterosynapse, whose plasticity involves multiple synaptic inputs, including one dendritic input and two regulatory terminals. Thanks to the rich ion dynamics of ionic/electronic hybrid devices, Di et al. [97] proposed a sodium alginate/graphene oxide hybrid-based electrolyte-gated indium tin oxide (ITO) hetero-dendritic neuron with a multi-gate configuration, as shown in Figure 6b. The BCM learning rule, as an advanced neural network learning rule, exhibits two characteristics, frequency-dependent synaptic plasticity and a shift in frequency threshold (θ_f_). By integrating coplanar gates, the team successfully modulated the BCM learning rule under the hetero-dendritic neuron model. The bottom gate (G_1_) of the device is regarded as the dendritic input, while the coplanar gates (G_m1_ and G_m2_) are considered the regulatory terminals. Figure 6c shows the θ_f_ value as a function of the V_m1_ amplitude and V_m2_ amplitude for Δt of 250 ms and 500 ms. When V_m1_ and V_m2_ are −0.1 V and 1.5 V, the θ_f_ values for Δt of 250 ms and 500 ms are approximately 30.7 Hz and 22.2 Hz, respectively. When V_m1_ and V_m2_ are 0.5 V and −2 V, the θ_f_ values for Δt of 250 ms and 500 ms change to approximately 15.1 Hz and 3.7 Hz. These results indicate that the synaptic weights of the device can be modified through the precise programming of inhibitory and excitatory stimuli at the regulatory terminals, thereby dynamically adjusting the BCM learning rule. He et al. [98] proposed a capacitively coupled multiterminal oxide-based neuro-transistor designed for spatiotemporal information processing. As shown in Figure 6d, this device features multiple in-plane gate electrodes oriented in various directions. In one direction, an electrode serves as the presynaptic input for a single dendritic branch, while electrodes in other directions function as presynaptic terminals distributed across the dendritic tree. As shown in Figure 6e, the ratio of the difference in EPSC peak amplitudes (Peak) evoked by different spatiotemporal pulse sequences to the EPSC peak amplitude (Peak_C1E2B3A4_) evoked by the spatiotemporal input sequences in the direction of C1→E2→B3→A4 (Peak/Peak_C1E2B3A4_-1) gradually decreases with the increase of the time interval between each stimulus. This result indicates that the device possesses the capability to recognize spatiotemporal input sequences from multiple dendritic branches. Additionally, as an example of spatiotemporal information processing, a simple artificial neural network with two input terminals and two output terminals was constructed, successfully simulating the sound localization function of the human brain, as shown in Figure 6f. This capacitively coupled multiterminal neuromorphic transistor demonstrates excellent spatiotemporal information integration capabilities. It should be noted that the sound localization simulation in this work utilized electrical signals instead of acoustic signals as external input and it only simplified the recognition of sound angles within the 0–180° range. Furthermore, the minimum time difference of 25 ms between two presynaptic pulses exceeds the interaural time difference found in biological brains, which may limit the application of this device in high-precision sound localization systems. Utilizing faster electrical testing platforms, electrolytes with higher ionic mobility, and coupling with more sensitive sound sensors will help overcome the aforementioned issues, thereby enabling multifunctional and high-precision sound localization.

Artificial neurons are the core of event-based neuromorphic sensors, which mimic the behavior of biological neurons to respond to specific events or changes in inputs by firing action potentials or spikes. This feature ensures that event-based sensors transmit and process information only when necessary, leading to a reduction in data bandwidth, latency and power consumption compared to traditional continuous sensing approaches. Although artificial neurons have been used in various advanced sensors for capturing events related to the human sensory system and in neuromorphic processors, some of these sensors and circuits have drawbacks, including rigidity, lack of flexibility, inability to sense ions and biomolecules, and poor biocompatibility. Therefore, continuous innovation and development at the material, device, circuit, and system levels are essential.

### 3.3. Neuromorphic Computing

**Figure 7:**
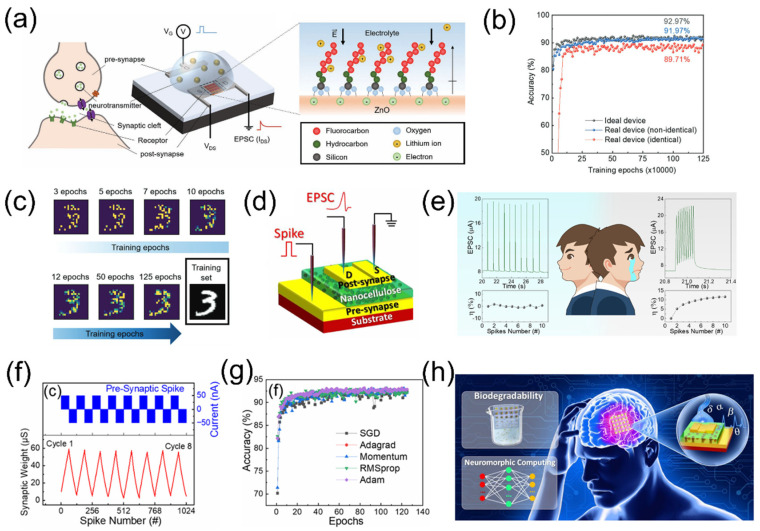
(**a**) Schematic illustration of a biological synapse and a Li^+^ electrolyte-gated PFOTS_5_/ZnO EGT. (**b**) The recognition accuracy of handwritten digits in simulations of a three-layer artificial neural network. (**c**) Visualization images of the accuracy of each epoch during the learning process in the simulation. Reproduced with permission [103]. Copyright 2022, Wiley-VCH GmbH, Weinheim, Germany. (**d**) Schematic of biodegradable nanocellulose-gated ITO neuromorphic transistors. (**e**) Schematic representation of human health status and its neurosensitization process under external stimuli. (**f**) Effective modulation of synaptic weights in the device by current pulses under eight cycles. (**g**) The accuracy of pattern recognition under different algorithms. (**h**) Schematic illustration of the potential application of biodegradable nanocellulose-gated ITO neuromorphic transistors in a brain–machine interface. Reproduced with permission [104]. Copyright 2023, American Chemical Society, Washington, DC, USA.

Neuromorphic computing was first introduced by Caver Mead in 1990 [99], with the aim of mimicking the structure and function of the human brain to achieve high-performance computing. Compared to traditional von Neumann systems, neuromorphic computing offers advantages such as faster processing speeds, lower energy consumption, and parallel processing capabilities, presenting significant potential for future AI applications, including smart healthcare, biological interfaces, and soft robotics [100,101,102]. As a novel computing architecture, neuromorphic computing focuses on creating electronic circuits or algorithms that can simulate the behavior of neurons and synapses, thereby enabling efficient data processing and learning capabilities. The approach of implementing neuromorphic computing at the synaptic/neural level has garnered widespread research interest. As a promising class of neuromorphic devices, EGTs are widely used to achieve functions such as synaptic emulation and neuromorphic computing. As shown in Figure 7a, Jin et al. [103] introduced a self-assembled monolayer (SAM) of fluorinated alkyl silane (FAS) as the channel–electrolyte interlayer in Li^+^ EGTs. Compared to EGTs without FAS, the FAS-treated EGTs, which contained five fluorinated alkyl chains (1H,1H,2H,2H-perfluorooctyltriethoxysilane, referred to as PFOTS_5_), significantly enhanced the retention of Li^+^ ions and the EDL effect. The Li^+^ electrolyte-gated PFOTS_5_/ZnO EGT exhibited excellent non-volatility, with an EPSC decay time exceeding 1800 s. Benefiting from the exceptional ion capture capability of the fluorinated alkyl chains, this EGT demonstrated outstanding multi-conductive states, high linearity, and symmetry, which are essential for performing neuromorphic computing. Based on the device characteristics of the PFOTS_5_/ZnO EGT, a three-layer artificial neural network (ANN) was constructed for recognizing a MNIST handwritten dataset. As shown in Figure 7b, recognition accuracy of ~89.71% with identical gate pulses and ~91.97% with non-identical gate pulses were achieved, which are close to the value of the ideal device of ~92.97%. Figure 7c shows the visualization images of the accuracy of each epoch during the learning process in simulation. These results indicate that PFOTS_5_/ZnO EGT is highly suitable for neuromorphic computing applications, offering potential for the construction of miniaturized, low-power, and efficient AI neural networks.

Brain-inspired neuromorphic computing and portable intelligent electronic products have also received increasing attention. Wang et al. [104] proposed biodegradable nanocellulose-gated ITO neuromorphic transistors, with the device structure illustrated in Figure 7d. The solution-processed nanocellulose-based solid-state electrolyte exhibits a high EDL capacitance of ∼1.5 μF/cm^2^ and a high proton conductivity of ∼1.4 × 10^−3^ S/cm at room temperature, leading to a low operating voltage of <2 V for the transistor. Benefiting from extremely strong proton gating effects, this device has also successfully emulated the pathogenesis of anxiety disorder (AD) at the hardware level. Figure 7e schematically illustrates the state of a healthy person and neurosensitization process under external stimuli. By rationally programming the voltage of 10 repeated presynaptic spikes (1.0 V, 10 ms), the channel conductance change ratio of the device can vividly mimic the induction, development, and attack processes of AD, i.e., “health status”, “neurosensitization”, “primary and secondary fear”, and “fear–adrenaline secretion–exacerbated fear”. Interestingly, the synaptic weight of the device can be regulated by current pulses. Figure 7f shows the conductance weight endurance of the device under eight cycles. In addition, a two-layer multilayer perceptron (MLP) neural network was established to recognize the MNIST handwritten dataset under different training algorithms. After 125 training epochs, the highest recognition accuracy of ~93.18% was achieved with the RMSprop algorithm, as shown in Figure 7g. The nanocellulose-gated ITO neuromorphic transistor has potential for use in “green” portable devices and brain–machine interfaces, as shown in Figure 7h.

EGTs exhibit excellent mechanical flexibility, low operating voltage, and a working mechanism similar to biological synapses. Significant progress has been made in hardware neuromorphic computing. However, the uniformity, compatibility, and stability of device arrays remain limited. Additionally, current research in neuromorphic computing primarily focuses on purely electrically controlled synapses and neurons, leaving substantial gaps in power consumption and functional applications compared to the human brain, which restricts the development of neuromorphic chips. Therefore, developing high-density technologies, designing more sophisticated device structures, such as vertical and three-dimensional channel structures, and preparing flexible and multifunctional high-density device arrays will be essential.

## 4. Bionic Perceptual System Based on EGTs

The prolonged evolutionary process has endowed biological organisms with complex sensory systems that possess unique advantages such as high parallelism, high fault tolerance, adaptability and low power consumption [105,106]. The sensory organs of biological organisms can detect and process external information, and transmit the processed signals to the brain for final information judgment, thereby providing vivid and efficient interaction with the real world. Inspired by the powerful signal processing capabilities of biological nervous systems, studies on neuromorphic electronics at the hardware level and bionic neuromorphic sensory systems are expected to achieve compatibility with biological neural signals, and thereby to construct intelligent and efficient sensory systems and human–machine interaction interfaces.

### 4.1. Mono-Modal Perception

**Figure 8:**
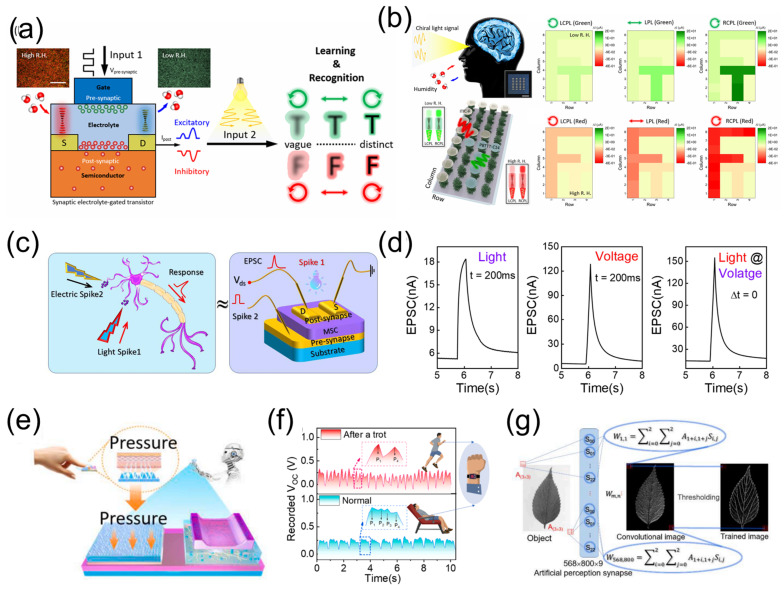
(**a**) Voltage pulses and light regulate EGT to mimic the learning and recognition processes in neuromorphic computing. (**b**) The synergistic effect of optical and electrical signals enables the realization of different colors and intelligent chirality recognition. Reproduced with permission [112]. Copyright 2023, American Chemical Society, Washington, DC, USA. (**c**) Schematic illustration of the optoelectronic synergy coupling of MPEC ITO neuromorphic transistors. (**d**) EPSCs triggered by a single optical pulse, a single electrical pulse and photoelectric synergy. Reproduced with permission [85]. Copyright 2023, Elsevier Ltd., Kidlington, UK. (**e**) A schematic diagram of a self-powered tactile sensing platform coupled with TENG and synaptic transistors. (**f**) Pulse wave signals collected by the TENG before and after exercise. (**g**) Tactile edge perception based on triboelectricity. Reproduced with permission [9]. Copyright 2023, Elsevier Ltd., Kidlington, UK.

A biological nervous system comprises the central nervous system and the peripheral nervous system. They work together to transmit and process sensory information while coordinating various bodily functions. For humans, five senses, i.e., touch, vision, taste, hearing, and smell, serve as the primary means of acquiring external information and play a crucial role in survival and communication. In recent years, inspired by human sensory organs, numerous neuromorphic devices and bionic sensors capable of converting various external stimuli, including light, sound, mechanical, and chemical inputs, into electrical signals have been extensively studied for the development of bionic artificial perceptual systems [107,108,109,110]. The eye is the most important organ for human perception. Approximately 80% information from external surroundings is acquired through vision. When light signals are detected by photoreceptor cells in the retina, the incoming light signals are converted into electrical signals and preprocessed to filter out excess visual data [111]. Inspired by this process, researchers have developed optoelectronic memory devices for artificial visual systems to achieve adaptive optical perception and exhibit visual memory behavior. As shown in Figure 8a, Tsukruk et al. [112] utilized a humidity-responsive bio-electrolyte layer to integrate a bio-synaptic transistor with balanced inhibitory and excitatory functions. In this system, the active layer of chiral cellulose nanocrystal (CNC)/PEG/NaCl composite films and optoelectrically modulated semiconductor conjugated channels work together to achieve responses on the polarization state of light and relative humidity. In the active bio-electrolyte-gated transistor (BEGT), the injection of charges from gate electrodes or mobile ions within the electrolyte material or the slow polarization of permanent dipoles modulates the backward current during transfer curves. Unlike a simple light–dark mode, this system can separate and transmit information related to different colors and chiralities to the occipital lobe of the central visual system. In addition, by applying different wavelengths and circular polarization of light, the optical synaptic array can not only distinguish between two colors (red and green) but also recognize the green letter ‘T’ and the red letter ‘F’ under varying polarization states. This capability mimics the environmental adaptation and selective memory functions of the advanced human visual system for future robotic vision, as illustrated in Figure 8b. This study not only advances the fundamental understanding of the optoelectronic properties of humidity-controlled synapses but also offers significant application potential for the development of next-generation bionic neuromorphic devices and neuro-robots.

Shi et al. [85] proposed an ITO optoelectronic neuromorphic transistor with a mesoporous silica coating (MSC) as gate dielectric, aiming to explore the light response capabilities of synaptic devices (Figure 8c). The synaptic response containing spatiotemporal information is crucial for the visual assistance learning and color recognition functions of the human eye. The oxide neuromorphic transistor can receive multiple spatiotemporal spikes from the outside world and perform spatiotemporal integration. As shown in Figure 8d, the absolute amplitudes of the EPSCs triggered separately by a light stimulus (0.9 mW cm^−2^, 200 ms) and an electrical stimulus (1 V, 200 ms) were 13 nA and 123 nA, respectively. However, when the light pulse and electrical pulse were synchronously applied, the EPSC absolute amplitude was ~139 nA, indicating that the device possesses spatiotemporal integration behavior for both optical and electrical stimuli. The emulation of these essential synaptic plasticity behaviors further enhances the potential of electrolyte-gated transistors in future neuromorphic engineering applications.

Humans perceive their surrounding environment through various sensory organs. Among these, touch serves as the most extensive perception system, providing contact information while also protecting the body from harm. Various receptors in the skin can detect mechanical, thermal, and electrical stimuli, eliciting a wide range of sensations. Tactile perception relies on an integrated process of sensing, refining, and learning, significantly shaping human interactions with the external environment [113,114]. Flexible electronic skin has been investigated to achieve tactile functionality and has found widespread applications in fields such as smart prosthetics, flexible robots, and human–machine interfaces [115,116]. Chen et al. [9] developed a neuromorphic self-powered artificial tactile pathway for emulating tactile perception by coupling a self-assembled single-electrode triboelectric nanogenerator (TENG) with a synaptic transistor on the single substrate, as schematically shown in Figure 8e. The TENG is integrated into the bottom gate of the transistor, allowing the gate bias control to be replaced by the output voltage (V_OC_) from the TENG. When pressure is applied to the TENG, an electrical signal is transmitted from the conductive layer to the gate, forming a control voltage. This sensory platform can detect external pressure information through the channel current of the synaptic transistor, enabling it to be used for detecting wrist pulse waveform signals. High-sensitivity pulse signals can further reveal characteristic information from the waveform, including the systolic peak (P_1_), reflected systolic peak (P_2_), dicrotic peak (P_3_) and end-diastolic pressure (P_4_). Figure 8f shows the V_OC_ pulses from the flexible TENG wrapped around the wrist before and after exercise. It is observed that the P_2_ and P_3_ peaks disappear in the pulse waveform after exercise, mainly due to increased heart rate, which causes the heart and arterial vessels to contract, thereby preventing the propagation of P_2_ and P_3_ in the peripheral arteries. These results indicate the significant potential of this sensory platform for health monitoring and rehabilitation training. Interestingly, this sensory platform enables a tactile-based image edge detection method, simulating the non-visual image generation function of the human brain, as shown in Figure 8g. This research provides new design insights for human–machine interaction perceptions, such as electronic skin and wearable monitoring devices.

### 4.2. Multi-Modal Fusion Perception

**Figure 9:**
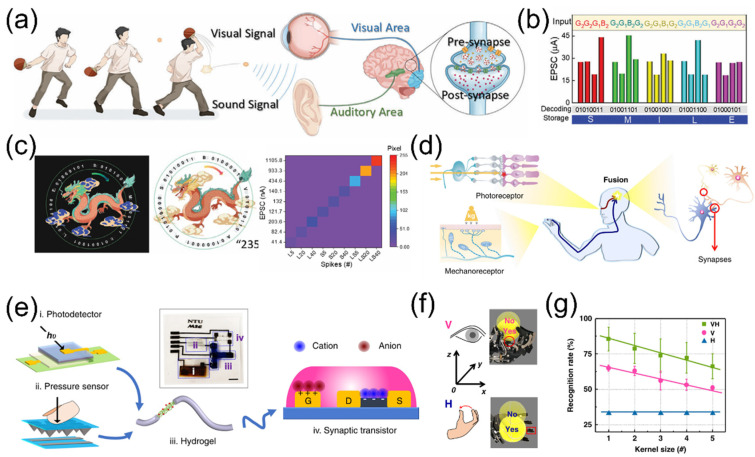
(**a**) A schematic diagram of a human visual and auditory collaborative perception system. (**b**) The BVAPS decodes the EPSC responses triggered by five sets of sound signals (i.e., G_2_G_2_G_1_B_2_, G_2_G_1_B_2_G_2_, G_2_G_1_B_1_G_2_, G_2_G_1_B_2_G_1_, and G_2_G_1_G_2_G_2_) into the word “SMILE” according to ASCII code. (**c**) Image visual decryption, decryption and normalized key pixel values under a visual–auditory fusion scheme. Reproduced with permission [118]. Copyright 2023, American Chemical Society, Washington, DC, USA. (**d**) Schematic diagram of visual–haptic fusion perception based on biological neural networks. (**e**) A bimodal artificial sensory neuron patch for visual–haptic fusion. (**f**) Visual–haptic fusion based on a bimodal artificial sensory neuron patch for motion control. (**g**) Visual–haptic fusion based on bimodal artificial sensory neuron for multi-transparency pattern recognition. Reproduced with permission [120]. Copyright 2018, WILEY-VCH Verlag GmbH & Co. KGaA, Weinheim, Germany.

The process of multisensory integration plays a crucial role in shaping cognitive, learning, and memory functions observed in human brain. The fusion of sensory information enhances environmental perception, facilitating tasks such as emotional regulation, scene memory, and learning. Visual and auditory information accounts for more than 90% of the total information processed by humans [19,117]. As shown in Figure 9a, external information is perceived and preprocessed by the eyes and ears, and then sent to the visual and auditory cortex for post-processing. The brain makes decisions and accumulates experience based on the complementary information from the two channels, enabling people to have a more comprehensive and in-depth understanding of the surrounding world. Given the significance of the human perceptual system, researchers are focusing on developing various electronic devices to construct bionic multimodal perception systems that are intelligent, efficient, and capable of simulating functions of living systems. Wang et al. [118] proposed a bionic visual–auditory perceptual system (BVAPS) featuring information encryption and decryption capabilities, achieved through the integration of chitosan-gated oxide ionotronic neuromorphic transistors and auditory sensors. Leveraging the strong interface ion/electron coupling effect of neuromorphic transistors, this system exhibits remarkable multi-modal sensory abilities on sound and light, demonstrating flexibility and efficiency across multiple application scenarios. The perception system encodes different sound information using ASCII codes, enabling encrypted transmission and decoding of audio information. As shown in Figure 9b, when a series of sound signals G_2_G_2_G_1_B_2_, G_2_G_1_B_2_G_2_, G_2_G_1_B_1_G_2_, G_2_G_1_B_2_G_1_, and G_2_G_1_G_2_G_2_ are loaded on the BVAPS, they can be decrypted into ASCII codes by corresponding EPSCs. Subsequently, the decrypted ASCII code information is demodulated into a single word “SMILE”, completing the encryption transmission and decryption reading of sound information. Additionally, the system is capable of encrypting and decrypting visual images through a visual–haptic fusion perception scheme, as shown in Figure 9c. These achievements pave the way for new possibilities in intelligent electronic systems with multimodal perception and information processing capabilities. Multisensory integration is a prominent feature of the brain, enabling better and faster responses compared to unisensory integration, particularly when single sensory cues are weak [119].

Visual and tactile cues are densely received and perceived during our interactions with the surrounding environment. These two cues are closely associated and interpreted in the inferior parietal cortex to provide supramodal spatial capabilities, as shown in Figure 9d. Wan et al. [120] developed a bimodal artificial sensory neuron (BASE) based on ionic/electronic hybrid neuromorphic electronics to implement the visual–haptic fusion. As shown in Figure 9e, this BASE collects optical and pressure information from a photodetector and a pressure sensor, respectively. It transmits the bimodal information via ionic cables and integrates it into the postsynaptic current using synaptic transistors. By synchronizing the visual and haptic feedbacks, sensory neurons can be stimulated at multiple levels, allowing for the manipulation of skeletal myotubes and robotic hands (Figure 9f). Furthermore, through simulating multi-transparency patterns recognition tasks, enhanced recognition capabilities achieved through integrated visual/haptic cues can be confirmed, as shown in Figure 9g. This bionic design holds potential for advancing robotics and neuromorphic system technologies by endowing them with supramodal perception capabilities.

It is noteworthy that extensive use of various materials, including two-dimensional materials, optoelectronic materials, organic polymeric materials, and biomaterials, in the fabrication of neuromorphic devices and bionic sensors has significantly advanced artificial perception systems designed to simulate biological synapses and neural system functions [121,122]. These systems possess the ability to perceive and process sensory information akin to neural pathways. They can not only detect various external stimuli but also convert them into electrical signals through artificial perceptual systems, thereby mimicking the somatic mechanisms in biological sensory nerves. Nevertheless, biological systems always outperform electronic devices in interacting with the real, dynamic world due to their advanced sensorimotor skills. Currently, multimodal artificial perceptual systems based on neuromorphic devices face numerous challenges. Existing research lacks supramodal perceptual capabilities that could enhance the reliability and accuracy of these devices, primarily focusing on individual artificial perceptual systems. Additionally, most current bionic perceptual research is concentrated on the sensing aspect, with limited studies on subsequent related driving systems. Therefore, developing multisensory artificial perceptual systems that align more closely with biological principles while maintaining low complexity and high energy efficiency is crucial for advancing artificial intelligence and neuromorphic computing.

## 5. Summary and Outlook

In recent years, brain-inspired neuromorphic engineering has emerged as a global research hotspot, positioned to be a key support and innovative driving force for the development of AI, potentially triggering a new wave of technological revolution. Neuromorphic devices based on hardware approaches have gained widespread attention due to their significant role in brain-inspired computing. Among them, EGTs have gained significant attention in the construction of bionic synapses and artificial neural networks due to their unique characteristics, including the ionic relaxation behavior of electrostatic modulation, low operating voltage, low power consumption, and multi-gate modulation capability. This review first introduces EGTs, focusing on two working principles, i.e., electrostatic modulation and electrochemical doping. Additionally, the selection of electrolyte materials and channel materials for EGTs are discussed. Based on the rich ion dynamics and unique conductivity adjustable characteristics of EGTs, their applications as bionic synapses and neuron devices in neuromorphic function simulation are discussed. Finally, the coupling of EGTs with bionic sensors and their applications in artificial perceptual systems are presented. Numerous studies have shown that EGTs have significant potential in wearable electronics, health monitoring, and human–computer interactions.

As novel memristive devices based on ionotronic coupling effects, EGTs with ionic conductor electrolytes as gate dielectrics exhibit significant potential in neuromorphic electronics. Owing to unique interfacial ionotronic coupling characteristics and associated electrochemical processes, EGTs enable diverse operational modes, providing a viable pathway for constructing energy-efficient, self-adaptive iontronic neuromorphic systems. With advancements in ultrafast ionic conductors and 3D heterogeneous integration technologies, EGTs are anticipated to catalyze transformative applications in AI, brain–machine interfaces, and flexible bioelectronics, bridging the gap between biological intelligence and artificial hardware architectures. Despite notable advancements in brain-inspired neuromorphic engineering, current EGTs-based neuromorphic electronics still face critical challenges as outlined below.

(1) The selection of electrolyte materials for EGTs is extensive. Yet, certain electrolytes suffer from degradation or aging during prolonged operation due to ion migration hysteresis and electrochemical reactions, compromising long-term device stability. Additionally, inherently slow ion transport dynamics in solid state EGTs, governed by ion mobility and diffusion rates, fail to match the sub-millisecond response of biological synapses, severely limiting their applicability in scenarios requiring rapid switching or high-frequency operation. To address these limitations, promising strategies involve the development of polymer–inorganic hybrid electrolytes that balance ion mobility with chemical stability. Compositional engineering, such as tailoring ion concentration, incorporating stabilizing agents, or employing mixed-solvent systems, offers a viable pathway to achieve composite materials with both high ionic conductivity and operational durability.

(2) Current fabrication techniques constrain EGTs to large device dimensions, typically above the hundred-micrometer scale, with their scalability remaining unverified. Furthermore, challenges in device uniformity persist, as conventional CMOS-compatible processes struggle to support high-density 3D integration of EGTs. To address these challenges, employing advanced nanofabrication techniques, e.g., self-alignment and photolithography, to lower feature sizes and improve device density is imperative, which may also significantly reduce power consumption. Additionally, adopting high-precision printing methods such as inkjet printing or transfer processes could enable large-area uniform fabrication of EGT arrays.

(3) Development of multimodal bio-inspired intelligent systems based on EGTs faces significant challenges in sensory integration. For instance, incorporating diverse sensing modalities requires the integration of multiple sensitive materials, which often suffer from interfacial incompatibility with EGT electrolytes. Furthermore, existing artificial perception systems exhibit limited versatility, with few capable of coherently merging multisensory inputs to execute complex fusion-driven tasks. Thus, synergistic integration of EGTs with artificial receptors (e.g., cochlear or olfactory sensors) or hybrid systems combining triboelectric/photovoltaic energy harvesters with EGTs could enable environment-adaptive, energy-efficient sensing. Such approaches may pioneer self-powered sensing systems that unify multimodal perception and energy autonomy, offering innovative pathways for next-generation intelligent artificial perception platforms.
